# The Relevance of Human Whistled Languages for the Analysis and Decoding of Dolphin Communication

**DOI:** 10.3389/fpsyg.2021.689501

**Published:** 2021-09-21

**Authors:** Julien Meyer, Marcelo O. Magnasco, Diana Reiss

**Affiliations:** ^1^CNRS, GIPSA-Lab, Université Grenoble Alpes, Grenoble, France; ^2^Laboratory of Integrative Neuroscience, Rockefeller University, New York, NY, United States; ^3^Department of Psychology, Hunter College, New York, NY, United States

**Keywords:** human whistled languages, dolphin communication, whistled speech, interspecies communication, whistle signal processing, Silbo, dolphin whistles

## Abstract

Humans use whistled communications, the most elaborate of which are commonly called “whistled languages” or “whistled speech” because they consist of a natural type of speech. The principle of whistled speech is straightforward: people articulate words while whistling and thereby transform spoken utterances by simplifying them, syllable by syllable, into whistled melodies. One of the most striking aspects of this whistled transformation of words is that it remains intelligible to trained speakers, despite a reduced acoustic channel to convey meaning. It constitutes a natural traditional means of telecommunication that permits spoken communication at long distances in a large diversity of languages of the world. Historically, birdsong has been used as a model for vocal learning and language. But conversely, human whistled languages can serve as a model for elucidating how information may be encoded in dolphin whistle communication. In this paper, we elucidate the reasons why human whistled speech and dolphin whistles are interesting to compare. Both are characterized by similar acoustic parameters and serve a common purpose of long distance communication in natural surroundings in two large brained social species. Moreover, their differences – e.g., how they are produced, the dynamics of the whistles, and the types of information they convey – are not barriers to such a comparison. On the contrary, by exploring the structure and attributes found across human whistle languages, we highlight that they can provide an important model as to how complex information is and can be encoded in what appears at first sight to be simple whistled modulated signals. Observing details, such as processes of segmentation and coarticulation, in whistled speech can serve to advance and inform the development of new approaches for the analysis of whistle repertoires of dolphins, and eventually other species. Human whistled languages and dolphin whistles could serve as complementary test benches for the development of new methodologies and algorithms for decoding whistled communication signals by providing new perspectives on how information may be encoded structurally and organizationally.

## Introduction

Beyond humans, only a handful of species are considered to be vocal learners: cetaceans (Lilly, 1965; Herman et al., 1984; Reiss and McCowan, 1993; McCowan and Reiss, 1995a, 1997; Tyack and Sayigh, 1997), birds [passerines, psittacines (Todt, 1975; Pepperberg, 1981), and hummingbirds (Baptista and Schuhma, 1990)], bats (Esser, 1994), elephants (Poole et al., 2005), pinnipeds (Ralls et al., 1985; Stansbury and Janik, 2019), and some non-human primates (Snowdon, 2009; Lameira et al., 2013; Takahashi et al., 2015). Of these, birds, some primates, cetaceans, and notably, humans as well, employ whistled signals to communicate at long distance. Whistle communication consists of amplitude and frequency modulated tonal frequency bands that last for a certain amount of time, and the relevant information that the whistles contain resists degradation due to propagation and reverberation (see, for example, Marler, 1955; Busnel, 1966; Jensen et al., 2012).

**Figure 1:**
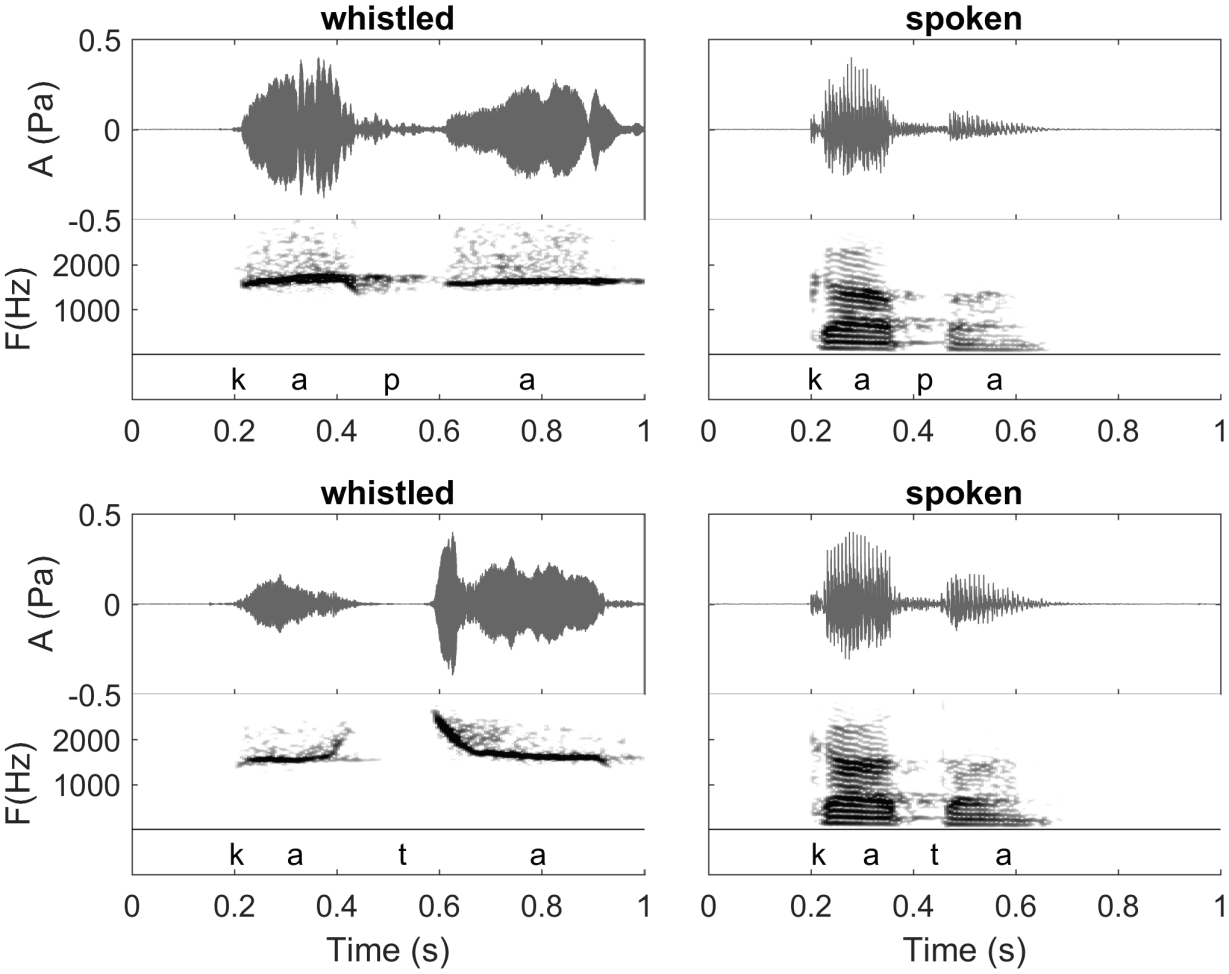
Waveforms and spectrograms of the words /kapa/ (Spanish orthography: “capa”; meaning: “hat” in English) and /kata/ (Spanish orthography: “cata”; meaning “tasting” in English) in spoken (right) and whistled (left) forms. All utterances are from the same speaker. Note the clear spectral difference between the two modalities (whistled *vs.* spoken). The common spectral dynamics between spoken speech and whistled speech can be observed clearly in the first /a/ because it was pronounced with more power than the second one in the spoken speech modality. The coarticulation of the initial /k/ with /a/ shows that for /ka/ whistled frequencies are close in shape to the dynamics of the first formant of spoken speech, whereas for the coarticulation /at/ whistled frequencies are close in shape to the dynamics of the second formant of spoken speech (see also “General description of dolphin and human whistled communication systems” on this topic; Recordings and edition by Julien Meyer, listen to sound extracts in Meyer and Diaz, 2021).

**Figure 2:**
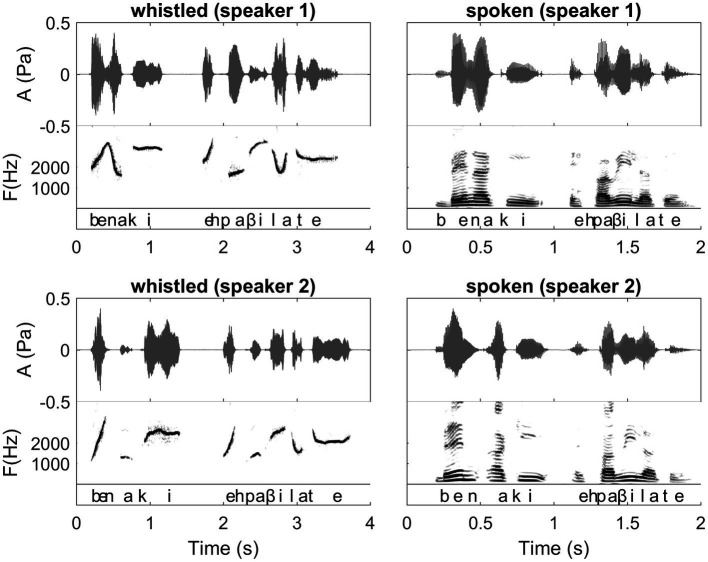
Waveforms and Spectrograms of the Spanish sentence “ven aqui espavilate” expressed both in spoken and whistled forms by two different speakers (presented in phonetic transcription in the figure and meaning “come here, hurry up”). In this figure, we can follow the dynamics of the sentence in both modalities. It illustrates the spectrographic differences/similarities between spoken and whistled modalities that are explained in “General description of dolphin and human whistled communication systems” of the paper. There is also a difference between speakers in the segmentation of the two first syllables corresponding to the boundary between the two first words “ven” and “aqui.” Interestingly, this difference happens in both spoken and whistled modalities showing that the speakers were consistent in their pronunciation across modalities. The first speaker says /bena/ (/n/ is continuous between /e/ and /a/), whereas the second speaker says /ben.a/ with a clear pause between /en/ and /a/. Both forms are acceptable for a Spanish listener. Other aspects that this figure illustrates also: 1) different whistlers whistle at different general frequency levels. 2) Vowels are distributed at different frequency levels and consonants represent modulations in frequency and amplitude of the more steady frequencies of the vowels. (Recordings and edition by Julien Meyer, listen to sound extracts in Meyer and Diaz, 2021).

Humans use different kinds of whistled systems, ranging from simple repertoires of codes for human-animal or human-human communication to the highly elaborate natural modality of speech commonly called “whistled language” or “whistled speech” (see reviews in Busnel and Classe, 1976; Meyer, 2015). In a human whistled language, the sender whistles syllables, words (Figure 1), and sentences (Figure 2) of the native human language, thus transposing spoken modal speech into a very different acoustic form to enable long distance dialogues as modulated whistles augment speech with properties of a real telecommunication system. This whistled modality of speech represents a natural and ancient human language practice often presented as a kind of natural ancestor for modern cellular phones. Whistled speech represents a more extreme transformation of the speech signal than shouting – the otherwise most universal natural adaptation of languages to speak from far. The transformation is much more drastic than what happens in other speech modalities, such as whispering and shouting, but the general principle of adapting speech for specific circumstances is similar.

In 1963 René Guy Busnel, the director of the CNRZ Laboratoire d’Acoustique Animale in France, presented a seminal paper at the First International Symposium on Cetacean Research entitled “Information in the Human Whistled Language and Sea Mammal Whistling” (Busnel, 1966). In this paper, he proposed there might be some advantage in comparing the whistles of dolphins with human whistled speech as the general form of the signals from both systems were quite similar, and by doing so, we might get an idea of the potential information carrying capacity of a frequency and amplitude modulated sine wave. Busnel’s research centered on the acoustic communication of animals and much of his work focused on the whistle communication and echolocation of dolphins and other cetacean species. Notably, he also published with his colleagues some of the first monographs and descriptions of human whistled languages of different regions of the world. Among these publications on the acoustic and phonetic characteristics of whistled languages was a first paper describing how Bearnese language was whistled in the French Pyrenees (Busnel et al., 1962), a second paper proposing for the first time a pluridisciplinary collection of studies on one whistled language, whistled Turkish (Busnel, 1970), and the first book synthesizing the extant knowledge on whistled languages with a worldwide vision (Busnel and Classe, 1976). At the time of these publications, whistled languages represented a little-known form of human acoustic communication and the linguistic aspects had been described only for a few populations (whistled Mazatec, in Mexico first reported by Cowan, 1948; and whistled Spanish (locally called Silbo) first described by Classe, 1956). The majority of prior research had been done in anthropology (e.g., Lajard, 1891; Eboué, 1935; Stern, 1957). Since then, research in this domain has advanced considerably in various domains linked to language production and perception (see Meyer, 2021 for a recent review).

**Figure 3:**
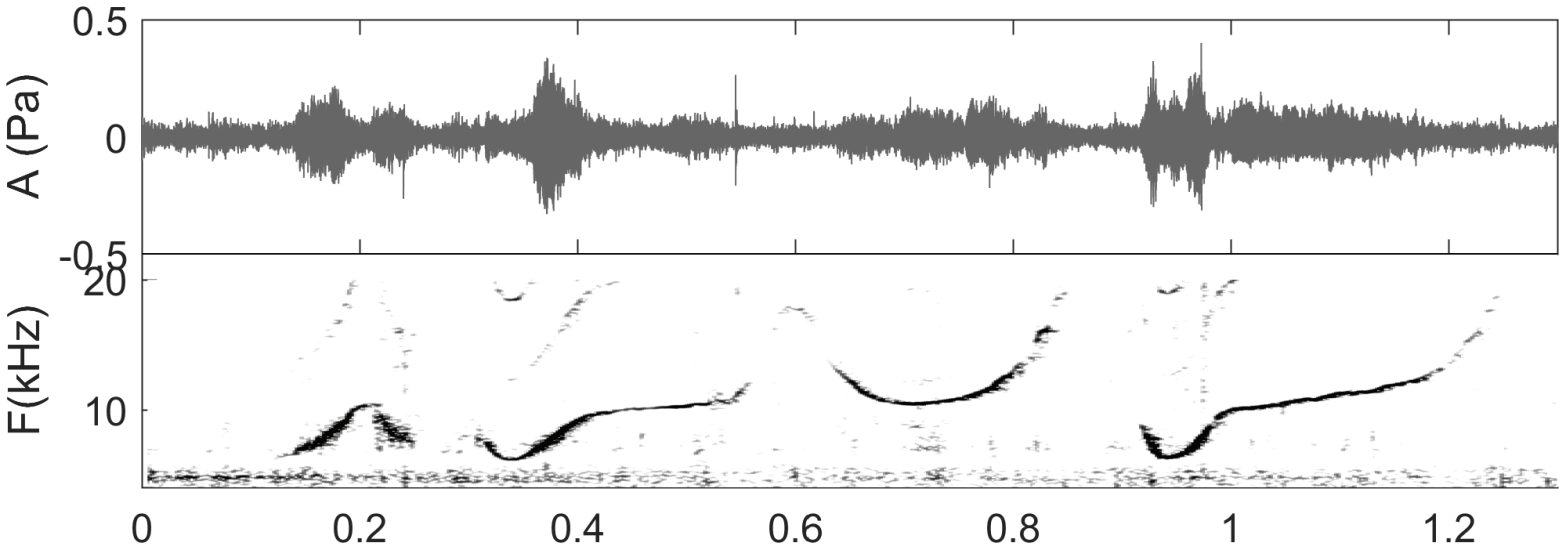
Spectrogram of a whistle sequence produced by a solitary female bottlenose dolphin at Turneffe Atoll, Belize, the field site of co-authors (MM & DR). (Unpublished data, recording captured by E. Ramos in the DR & MM collaborative research program, listen to sound in Reiss and Magnasco, 2021).

**Figure 4:**
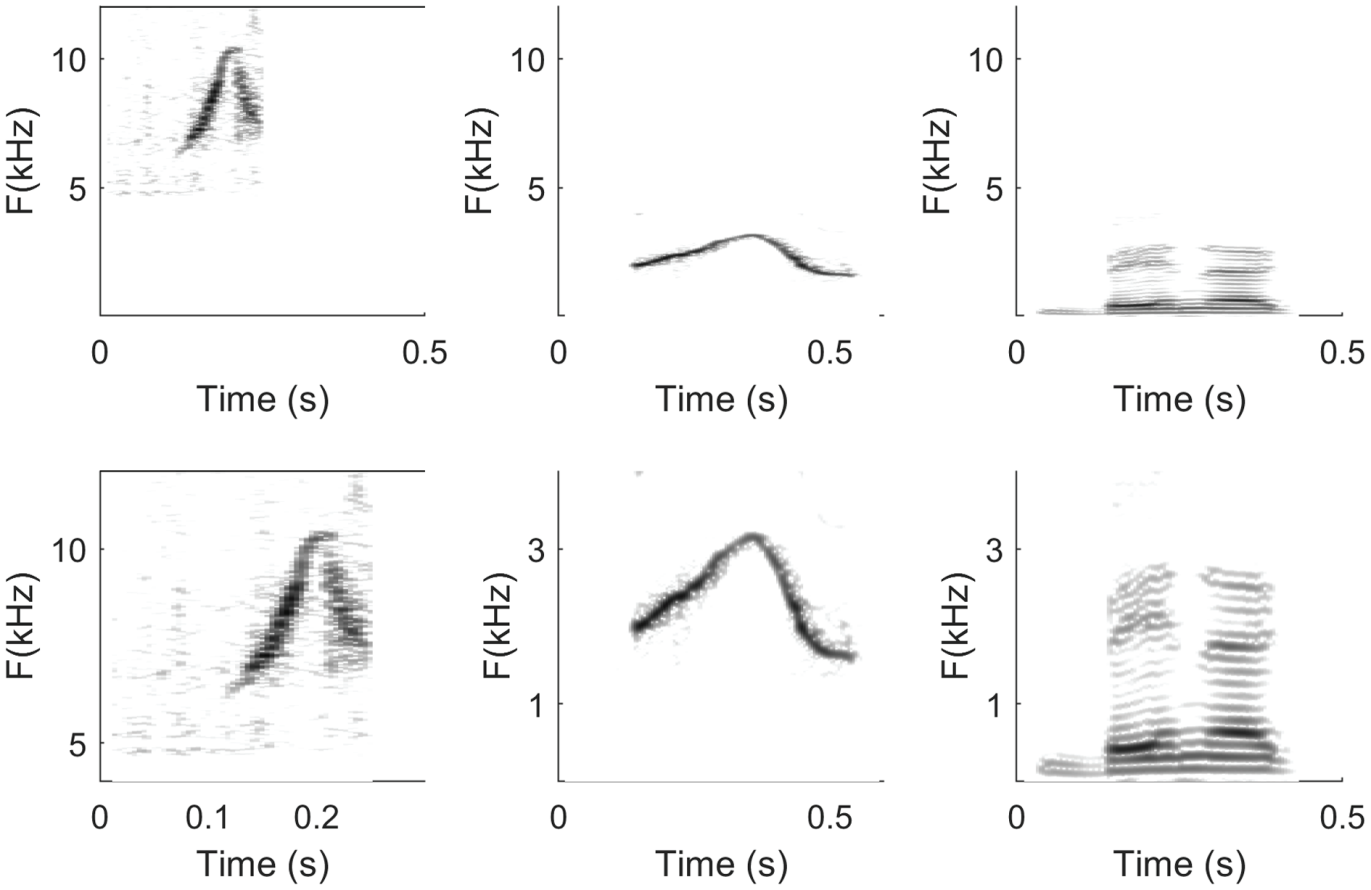
Spectrograms of a dolphin whistle (left column), a human whistled utterance (/bena/; middle column), and a spoken utterance (/bena/; right column) represented at two different scaling levels (upper line *vs.* lower line). The first row highlights differences in signal types (dolphin whistle *vs.* whistled speech *vs.* spoken speech), whereas the lower row highlights well similarities in the relative variations of frequencies. Scales in the upper line have the same x and y (for all three columns). In the lower row, the representation is zoomed differently in x and/or y according to the most representative frequencies of each signal type.

Of course, human and dolphin whistled communication systems differ in many respects: e.g., production mechanisms, the nature of the information encoded, the ecological milieu, and medium in which they are used (air vs. water). At the purely acoustic level, each species shows particular variations in the frequency range or the degree of frequency and amplitude modulation (see Figure 2 for a human whistled sentence, Figure 3 for a dolphin whistled sequence, and elements of comparison in Figure 4). However, some similar acoustic dynamic parameters and the functional use of whistles in natural surroundings provide biological and ecological relevance to compare these otherwise very different communication systems [in accordance, among others, with the sensory drive hypothesis (Endler, 1992) and the social complexity hypothesis (Freeberg et al., 2012)]. Humans and dolphins have in common to be highly encephalized social species, which live in fission-fusion groups, with culturally learned communication that includes acoustic signals encoding a great deal of information (Marino et al., 2007; Meyer, 2021). Notably, for both humans and dolphins, whistled communication has evolved in response to common socio-environmental pressures for communication over long distances in natural surroundings (see next “Socio-environmental parallels in the study of dolphin and human whistles”).

In this paper, we will provide background and description of human whistled languages and dolphin whistle communication and present relevant findings from the analysis of human whistled languages that may be helpful if applied to the analysis of dolphin whistle communication. We will also review two previously published experimental studies with dolphins (Batteau and Markley, 1967; Reiss and McCowan, 1993) and discuss the relevance and implications of their findings to the analysis of dolphin whistled communication. More specifically, we propose that our understanding of how phonetic information is encoded and represented in human whistled languages can provide insights and may correct what may be our human biases in how we parse, segment, and categorize dolphin whistles. We argue that such an approach may have important implications when looking for possible structures in dolphin whistles. Comparisons of the way whistled signals and signal sequences by humans and dolphins are visually perceived in spectrograms and categorized during the process of acoustic analysis of signals can offer new insights into acoustic boundaries and the organization and combination of elements.

Please note that in this paper, we use the phonemic transcription to represent the sounds as they are categorized in the mind of human speakers; − they typically appear between slashes “/.” This choice enables us to use the same symbols to represent both the spoken form and their altered transformations into whistles. In the figures only, we will use the phonetic transcriptions corresponding to the detailed pronunciation of a word in modal spoken speech which is the reference that is tentatively transposed by whistled speech. We sometimes also use the common English or Spanish orthographies, when convenient, and they appear between “”.

## Socio-Environmental Parallels in the Study of Dolphin and Human Whistles

Regarding human whistled languages, there are currently approximately 80 low density and remote populations around the world that are known to have adapted their local language to this particular whistled modality, using it for long distance communication (Meyer, 2015). Around 40 of them have been studied and/or recorded. One of the most striking aspects of the worldwide distribution of whistled languages is that they are found almost exclusively in association with certain types of habitats: mountainous topography and highly vegetated landscapes. Such ecological milieux has in common to create favorable conditions for long distance communication in rural settings because dense vegetation and rough topography often lead to physical isolation and constrain spoken communication (Wiley and Richards, 1978; Meyer, 2015, chap. 6) while applying selective pressures on long distance signals (Morton, 1975). Likewise, worldwide there are approximately 90 known cetacean species, 40 of which are extant dolphin species. Dolphins use multi-modal signals, whistles, other acoustic signals, and behaviors to communicate during social interactions and for long range communication (Marino et al., 2007; Luís et al., 2021).

Among humans, whistling the spoken language has developed as a means of coping with the frequent need of individuals to communicate from far distances and this mode of communication has facilitated the organization of everyday life when ordinary modal or shouted speech forms are inadequate, particularly during traditional subsistence activities (such as shepherding, hunting/fishing, and hill agriculture) that require frequent exchange of vital information. We know very little about the origin of human whistled speech practice, which is still a matter of supposition (just like for the origin of language). The fact that nowadays whistled speech mostly functions for long distance communication associated with activities involving the acquisition or supplying of food or other forms of subsistence, in several rural populations on all inhabited continents of the planet, fuels the hypotheses, and sometimes unites them with the theories about language origin (Meyer, 2015, chap. 9).

Human whistles in whistled speech are powerful and clear signals, which propagate well despite natural ambient noise and physical obstacles: they allow high power in a frequency band of 800 to 4000Hz corresponding to the best human audibility and sound discrimination (Schneider and Trehub, 1992), they limit signal masking due to a narrow bandwidth, and they fall higher than the frequencies where natural background noise is usually the most powerful [most noise from abiotic sources has energy below 1kHz, even if rivers, torrents, waterfalls, and sea rumble are important exceptions because they show strong amplitudes in the entire frequency spectrum (much as does white noise; Busnel and Classe, 1976; Meyer et al., 2013). Moreover, some species of birds, anurans, insects, and mammals produce songs that use the same bands of frequencies as human spoken and whistled speech, but they are much more intermittent, seasonal, and/or more biotope specific (Meyer, 2020)]. These properties define a real telecommunication system adapted to natural acoustic conditions that enables interlocutors to speak from far with birdlike sounds at distances typically going from around 50m to 2km depending on the place, with common median uses of 400–500m. For dolphins, sound travels particularly well underwater and is a very effective sensory signal for sensing distant stimuli in the marine environment (Jensen et al., 2012). For example, Jensen et al. (2012) employed a calibrated GPS-synchronized hydrophone array to record the whistles of bottlenose dolphins in tropical shallow waters with high ambient noise levels and reported median communication ranges of 750m and maximum communication ranges up to 5.74km.

Another important parallel between human whistled speech and dolphin whistles is that the nature of both have long remained a mystery for the general public and for science. In the case of whistled speech, this is largely due to the fact that it requires special training to be understood. It can easily be mistaken for a non-linguistic code and people who are not trained in whistled speech perception do not readily recognize words in their whistled form, even if they are fluent speakers of the language being whistled. Thus, whistled human language long remained little known and mysterious outside the communities practicing it because it was rarely identified as a speech act by researchers and travelers, and it is still sometimes the case where it has not been turned into a tourist attraction (Meyer, 2015). Another reason whistled speech remained unknown for so long in many regions of the world is that this practice survives only in some of the most remote forests and mountains of the planet and mostly among rural populations speaking some of the less spoken and less documented languages. Finally, even for linguists, its veracity as a language is hard to apprehend at first contact because of the elimination of canonical acoustic correlates of phonemes from the spectrum. This explains why the classical phonetic annotations fail to characterize such a dramatic change in production. Importantly, and thus worth noting, a drawback was that there has been resistance by linguistics in perceiving and acknowledging human whistle language as a true linguistic form up to the middle of the 20th century (see Busnel and Classe, 1976; Meyer, 2015) due to the perceived simplicity of the whistled signals. However, it shows the same design features as spoken speech including the principle of duality of patterning (Hockett, 1960). It is even a challenging opportunity for research that whistled speech produces a different perception of a fully intelligible sentence because this change can be interpreted as an example of “perceptual insight” or pop-out of a top-down perceptual process (Meyer et al., 2017) produced by higher-level knowledge and expectations concerning sounds that can potentially be heard as speech [much like what happens in artificial Sine Wave Speech (see Remez et al., 1981; Davis and Johnsrude, 2007) given that whistled speech relies on a more drastic and natural reduction to only one sine wave].

Concerning dolphins, since the 1960s, there has been an increasing scientific effort and interest in investigating and deciphering the whistle repertoires of bottlenose dolphins. This species has been the focus of many efforts due to the fact that this is the primary dolphin species that has been in aquaria which has afforded the opportunity to observe and record their acoustic signals and concurrent behavior. Bottlenose dolphins have encephalization levels second only to modern humans (Marino, 1998), show advanced cognition including the capacity for mirror-self recognition (Reiss and Marino, 2001), live in complex fission-fusion structured social groups, and show high levels of cooperative behavior in foraging, care of young, and mating (see review in Marino et al., 2007). As previously stated, dolphins are vocal learners. Through training, they have demonstrated the capacity to vocally imitate novel sounds, learn the concept of vocal imitation (Penner, 1966; Herman et al., 1984), and demonstrate the capacity to learn to comprehend human gestural “sentences” and “grammatical” rules (Herman et al., 1984). In the absence of explicit training, dolphins have exhibited an impressive proclivity for spontaneous vocal imitation of species-specific sounds (Tyack, 1986) as well as spontaneous imitation of novel computer-generated whistles and productive use of their facsimiles in behaviorally appropriate contexts (Reiss and McCowan, 1993; Hooper et al., 2006). The social complexity of dolphins has been described as more complex than that of any non-human (Conner, 2007). For example, bottlenose dolphins in Shark Bay, Western Australia have shown second and third order alliance formations and interactions that likely require complex social cognition for the recognition of individuals and regulation of interactions between alliance members across different contexts (Connor et al., 2011). The social complexity hypothesis (Freeberg et al., 2012) advances the idea that complex social systems require more complex communicative systems to regulate interactions among individuals. Consistent with this hypothesis, dolphins are likely to have evolved a complex communication system. Thus, dolphins have been and continue to be among the most promising non-human animals for studies of complex or language-like communication competencies and there has been much interest in deciphering dolphin whistled systems. Such studies afford us a unique opportunity to document and understand the structure and function of whistle communication in another large brained social mammal with profound differences in morphology, ecology, and evolution.

## General Description of Dolphin and Human Whistled Communication Systems

### Human Whistled Languages

A human whistled language is in reality a natural type of speech, in which whistlers transpose spoken speech into whistles, word by word, and even syllable by syllable. This mode of speech is always based on a spoken language. Whistled Spanish or whistled Turkish is still Spanish or Turkish expressed in a different sound modality (Busnel, 1970; Busnel and Classe, 1976). The procedure at play shows some similarities with other natural speech types, such as whispered or shouted speech, in which speakers also transform/modify the phonetics of speech at the source to adapt to special circumstances of communication. While shouted speech reinforces particularly voiced sounds of spoken speech, whistled speech transposes some spoken sounds to imitate them in whistles and the vocal folds do not vibrate anymore but just participate to control the air flow (Meyer, 2020). Just as in whispered and shouted speech modalities, the whistled speech productions always refer to the phonetics of spoken speech from which they maintain and augment some acoustical features while degrading others, enabling the listeners to still reconstruct cognitively the words and their meaning because the selected salient features are enough to recall the linguistic system of the language they speak (Busnel and Classe, 1976; Meyer, 2015). A notable difference of whispered and shouted modalities with the whistled one is that the latter requires special training to recognize speech sounds in whistles.

**Figure 5:**
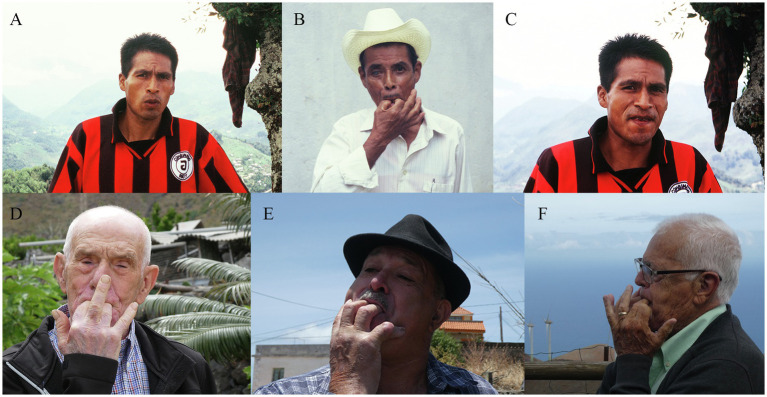
Some examples among the many different whistling techniques used for whistled speech worldwide. **(A)**. bilabial; **(C)**. linguo-dental; **(B**,**D-F)**, with two fingers in the mouth and forming a V shape at the contact of pressure with the tongue. Photos **(A-C)** represent three different techniques shown by Mazatec whistlers of Mexico (from Meyer and Diaz, 2017). Photos **(D-** **F)** represent three different techniques shown by some of the last traditional whistlers of El Hierro, Canary Island (Spain): the two techniques **(D**,**E)** are very characteristic of this island [Photos **(D-** **F)** courtesy of Julien Meyer (© Julien Meyer. All Rights Reserved)].

Whistled speech production by human whistlers is generated by a stream of compressed air in the mouth that is molded by the tongue, the jaw, the lips, and eventually the fingers (Figure 5). The articulation is more constrained than in the spoken form because talking whistlers need to sustain a whistled sound source while pronouncing the words (Meyer, 2015, chap. 5). A single person can use several techniques (Figure 5), but some of them are more frequent in each place, depending on the most common distances of communication. Techniques associated with very long distances of communication generally employ fingers in the mouth to accelerate the air stream and increase amplitude levels. The range of frequencies used by a whistle depends on the whistling technique used, of the power put into the whistle and some personal physiological characteristics, such as the size of the vocal cavity and dentition.

### Dolphin Whistle Communication

**Figure 6:**
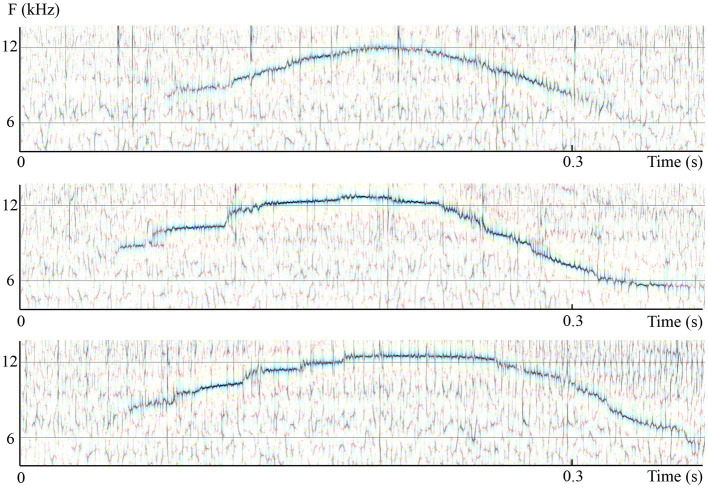
High-resolution consensus reassigned spectrograms of sound data captured at open sea in Bimini from wild Atlantic spotted dolphins shows three individual elements or up/down frequency modulations of a dolphin whistle presented as three subpanels. Notice the many subtle differences between them. Chief among them is the breaking of the second and third element into “steps,” which causes the whistle, if played back at a substantially reduced speed and pitch, to sound more like little “arpeggios” than smooth glides. Panels display multiband-wavelet reassigned spectrograms: temporal resolution, 12pixels/ms; frequency resolution, 1/40th of a semitone per pixel. (Recordings and edition by Marcelo Magnasco, listen to the three sound extracts as they were recorded in the wild in Bahamas. The original recording available online in Reiss and Magnasco (2021) is 1.5s long and contains the 3 whistles represented in the high-resolution spectrograms and multiple clicks and a squawk of *Stenella frontalis*).

**Figure 7:**
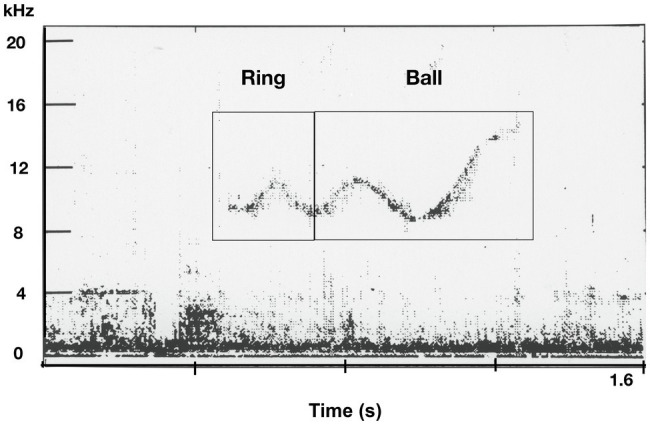
Spectrogram of an apparent combination whistle spontaneously produced by a bottlenose dolphin. The initial element resembles the computer-generated ring whistle and the end of the whistle resembles the computer-generated ball whistle. (from Reiss and McCowan, 1993).

Vocal signaling is a primary modality of communication in dolphins and other cetaceans although they communicate using a mixture of multimodal non-vocal signals acoustic that may be used alone or in conjunction with other forms of multimodal non-vocal signals during social interactions (see reviews in Herman and Tavolga, 1980; Popper, 1980; Herzing, 2015). Bottlenose dolphins produce a wide range of vocal signals that have been broadly characterized as narrow-band tonal or frequency modulated calls termed whistles (see Janik and Sayigh, 2013 for review), echolocation clicks (Au et al., 1974), and a wide variety other wide-band pulsed calls. It has been reported that dolphin whistles are produced by “pneumatically induced tissue vibrations,” similar to the way terrestrial mammals use their vocal folds and birds use their syrinx (Madsen et al., 2012). Madsen et al. (2012) analyzed whistle production of a dolphin breathing Heliox and found a lack of frequency shift in the dolphin’s vocalizations supporting the view that sounds are produced with tissue vibrations. Madsen et al. suggest that in this way, whistles can be effectively transmitted into the water without shifts or variability in frequency due to changes in water depth, air density, and recycled air volumes – thus preserving information, such as, possibly, individual identity conveyed in whistles. There is evidence that dolphins may have the ability to control the amplitude of their signals (Richards et al., 1984) as well as precise changes in frequency (Reiss and McCowan, 1993, and see Figures 3, 6, 7).

As previously mentioned, dolphins are vocal learners and variation in the acoustic repertoires of dolphins is thought to be due in part to social learning, other socio-ecological factors, and geographic isolation. For example, a recent comparison of acoustic signals recorded in nine dolphin populations in the Atlantic Ocean and the Mediterranean Sea indicated that all nine populations had rich and varied acoustic repertoires that included the same four broad categories of signals: whistles, burst-pulsed sounds, brays, and bangs (Luís et al., 2021). Much past and current research has focused on the dolphins’ production and use of signature whistles, defined as individually distinctive whistles thought to convey and broadcast the sender’s identity to other members of the social group [Caldwell and Caldwell, 1965, see review by Janik and Sayigh, (2013)]. Signature whistles have been reported as stereotypic whistle contours that are often produced in repetition or of loops of whistles and which comprise ~70–90% of whistles emitted by individuals (Caldwell and Caldwell, 1965; Tyack, 1986; Janik et al., 2006). From a broader perspective, vocal exchanges of whistle have been well documented in a variety of contexts including mother-calf exchanges during separations and reunions (Tavolga and Essapian, 1957; Smolker et al., 1993; McCowan and Reiss, 1995a) and the coordination of behavior (Moore et al., 2020) and appear to function in the maintenance of group cohesion (Janik and Slater, 1998). Dolphins have also been shown to imitate each other’s signature whistles and hypothesized to function as a label or name for other members of the group (Tyack, 1986). Whistle sharing and whistle convergence, the use of shared but individually distinctive rise-type signature whistles (contact calls) by dolphins in isolation, within and across social groups in aquaria have also been reported (McCowan and Reiss, 1995a,b, 2001; McCowan et al., 1998). It was also reported that social familiarity, one’s social group, and affiliations within the group may influence whistle acoustic structure in females (McCowan et al., 1998; Smolker and Pepper, 1999). Free-ranging adult male bottlenose dolphins have been reported to use similar whistles as they develop affiliative social relationships (Smolker and Pepper, 1999; Watwood et al., 2004) providing further evidence of whistle sharing and a convergence of whistle use in social groups. In terms of other aspects of the dolphin whistle repertoires beyond signature whistles, the use of other shared whistle types within social groups has been documented (McCowan and Reiss, 1995b; Jones et al., 2020).

## Considering Segmentation in the Whistles and Other Signals of Different Species

**Figure 8:**
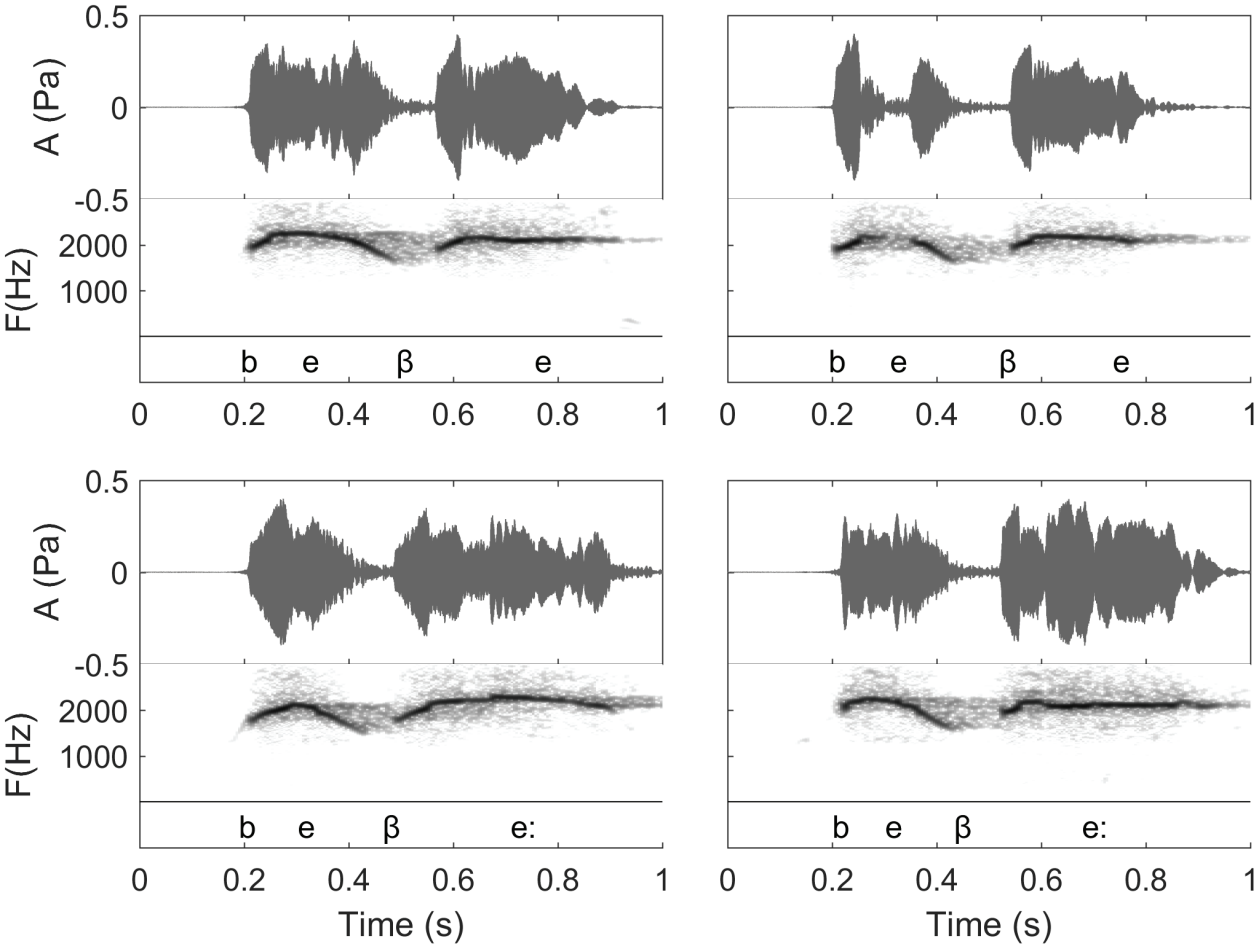
Waveforms and spectrograms of the Spanish words “bebe” (top, meaning “he drank”) and “bebé” (bottom, meaning “baby”). In the figure, the transcription shows the details of the spoken phonetic pronunciation (note that the symbol “:” signals a long vowel. Moreover, the fact that the second “b” of these two words bears a different phonetic symbol than the first one shows a particularity of spoken Spanish that is transferred to whistling). Each word is whistled twice by the same whistler. In this figure, we thus can see differences between different repetitions of a same word that are accepted by the proficient listeners, as well as the influence of relative duration of the two vowels of these words. In “bebé” (bottom), the second syllable is longer than the first one. Note that the general duration of the words is the same because all these words are whistled in the same conditions, but for “bebé”, the first syllable is shortened in comparison with the one of “bebe” (Recordings and edition by Julien Meyer, listen to sound extracts in Meyer and Diaz, 2021).

How do we segment, categorize, and determine acoustic structure in whistle sequences produced by humans and dolphins? How may information be encoded temporally in these whistle sequences? A profound difference presently exists in how we study and describe human vs. non-human animal communication. This principally comes from the particular insight we have regarding human languages. Notably, for example, studies of human speech include the abstract notions of linguistic syllable and phoneme. For linguists, syllables are language-dependent “building blocks” of words. The syllable is described in language sciences as a unit of organization for a sequence of human speech sounds typically made up of a syllable nucleus (most often a vowel) with optional initial and final margins (typically, called consonants). Speech sounds make up the surface acoustic form (or phonetic realizations that may be described in terms of consonants and vowels) of phonemes. Phonemes are small abstract underlying units considered to constitute minimum mental categories and representations for segments of words that can distinguish one word from another in a definite language. For example, the phoneme /b/ in Spanish represents a group of different phonetic realizations: It is pronounced in a significantly different way – and thus not represented phonetically by the same symbol – if it is in the beginning of a word or in between two vowels (as illustrated in Figure 8). The “phonotactic” rules of each language explain which sounds are allowed or disallowed in each part of the syllable. For example, English allows syllables beginning with a cluster of up to three consonants (as in the word “string”), whereas many other languages are much more restricted in the number of initial consonants in a syllable (such as Japanese or Spanish). Depending on the consonants between two vowels, the speech signal may be interrupted or not (for example, stop consonants interrupt completely the air stream, as shown in Figures 2, 10). Finally, the speech rate and the speech groups (often related to breathing) also influence speech segmentation (see for example Figure 2). To properly segment the signals, we hear in a human language, it is necessary to have competencies in this particular language. Our approach to speech segmentation would be biased if we determined word or syllable boundaries only on intervals of silence preceding or following an element. This is a very different view from the term “syllable” as used in bioacoustics, mostly in bird communication studies (e.g., Geberzahn and Aubin, 2014) where it is used in a purely acoustic sense: corresponding to a sound preceded and followed by silences.

This brings into question the relevance of how we traditionally segment and subsequently categorize whistles of other species in most bioacoustics studies, all the more as there is growing evidence of cases of merging and splitting of calls in the animal kingdom (Hailman and Ficken, 1986; Arnold and Zuberbühler, 2006; Suzuki, 2014; Engesser et al., 2015, 2016, 2019; Hedwig et al., 2015; Pardo et al., 2019). Strikingly, for example, in a recent paper investigating the process of vocal imitation and song development in Zebra finches, Tchernichovski et al. (2021) reported such merging or splitting of syllables by pupils when exposed to tutor songs. While the bioacoustics definition of syllable is an effective tool for measuring the dynamics of the signal, it may miss the sequencing due to species-specific perceptual processes.

The analysis of human whistled speech signals highlights the pertinence of this question in an original way due to the close resemblance of human whistled speech signals with the whistles of several animal species and to the particular insight provided by speech segmentation in syllables or speech sentences. One of the critical features of human language is duality of patterning (Hockett, 1960) which underlies the productivity of language. This property enables combinatorial structure on two levels: meaningless sounds (phonemes) can be combined and recombined into meaningful morphemes and words, which can be combined and recombined to form an infinite number of phrases and sentences. This may not be unique to human language as evidence for simple combinatorial structure has been already reported, for example, in the vocalizations of putty-nosed monkeys (Arnold and Zuberbühler, 2006) or of birds species, such as Chestnut-crowned babbler (Engesser et al., 2015, 2019) and southern pied babbler (Engesser et al., 2016). However, reliance on audible acoustic cues and visual inspection of spectrograms has influenced and somehow limited how we perceive and categorize animal communication in general and dolphin whistles in particular, including their temporal organization. Moreover, because little is known about how dolphins organize and structure their whistles we do not know where to draw the boundaries. This has been under-investigated in past studies due to the inherent difficulties in determining how dolphins psychoacoustically parse and organize what they hear and produce.

With dolphin whistles and human whistled speech signals, we are in a unique situation where we can compare two long distance communication systems based on whistles in two large brained social species. In human languages, speech segmentation is precisely a psychoacoustic process that requires a language competent listener to segment and parse a continuous or semi-continuous stream into meaningful speech. With whistled speech, which is the most studied of the speech surrogate practices, we have a promising opportunity to observe that it is possible to encode complex information in signals which look simple and to explore in details how it is encoded and organized in time. Importantly, we will see in the next sections that this alerts us to the fact that subtle and small transient changes or features in whistles may also encode key information in dolphins. The objective is not to infer that whistled communications of dolphins or other species encode the same type or the same complexity of information. Rather, we argue that a better understanding of how information is acoustically encoded in human whistled languages, which may appear as simple non-linguistic calls when represented in spectrograms, can provide insights as to how information may be encoded in the whistled communication of dolphins and other whistling species. Looking more closely at the details will obviously require using other analysis approaches than just using silent intervals between elements as indicators of meaningful boundaries.

## Human Whistled Speech Acoustic Structure: Encoding and Perception

Whistled speech profoundly modifies the phonetics of modal speech, applying a reduction at the source and in the frequency domain: the complex, multidimensional frequency spectrum of spoken speech is transformed into an imitation based on a simple unidimensional variation of the whistle (Figure 1 provides illustrations of this reduction on two Spanish words and Figure 2 provides an example on one Spanish sentence spoken and whistled by two different whistlers).

During whistled speech production, certain phonetic details present in modal speech are inevitably lost due to the simple melodic line encoding the words. However, to efficiently exchange messages with such whistles, there is a functional need of maintaining sentences intelligible, which is achieved by emulating/transposing salient phonetic cues of the language, as just explained, but also by choosing sentences that fit well to the context of the communication. Interestingly, it has been measured that whistled sentences remain intelligible to trained speakers in spite of the drastic frequency simplification at play (for a review, see Meyer, 2015, Ch. 8). For example, in whistled Turkish, Busnel and colleagues showed that isolate words are recognized at a rate of approximately 70%, whereas common whistled sentences are recognized at a rate of approximately 80–90% (see Busnel, 1970; Moles, 1970). As we will see in the next paragraph, intelligibility rates are also dependent on the tonal/non-tonal structure of the language that is transposed. But anyway, the combination of abridgment and sound iconicity highlights some salient features of the language through which the interlocutors can cognitively reconstruct the meaning of the message based on many of the same types of contextual cues as in human modal speech (phonetic cues of neighboring phonemes, words, and sentences, but also thema and rhema). For example, listeners show “phonemic restoration,” the filling in or reconstructing of missing or ambiguous sounds within a word or words within a sentence (Conway et al., 2010).

The significant diversity of whistled languages that have been found enabled research to highlight that whistled transpositions of speech conform differently to some essential aspects of language structures because languages of the world use different sets of sound inventories to code their linguistic systems and because whistling adapts to these sets and to the rules of organization of sounds that characterize each language. This naturally results in a language-specific selection of some of the salient features of consonants, vowels, and/or tones of a given language. Two main strategies of transposition in the spoken-to-whistled substitution have been observed, depending on a major typological distinction: tonal vs. non-tonal languages (see details on this distinction in Busnel and Classe, 1976; Rialland, 2005; Meyer, 2015).

In tonal languages – in which the spoken pitch can change the meaning of a word – whistles transpose primarily the phonemic tones carried by the acoustic pitch of the voice in each syllable and their specific modulations/contours within or between these linguistic tones (“pitch whistling” strategy). Thus, the possibility of transmitting complex sentences to a trained listener depends on the informational load carried by tonal prosody in the language. For this reason, in tonal languages with low informational load on tones (such as Surui of Rondônia), whistled sentences are more formulaic and therefore more predictable, while high levels of intelligibility can be reached in unpredictable sentences in tonal languages with rich tonal systems for lexical load, such as Hmong (Meyer and Gautheron, 2006) or Chinantec (Sicoli, 2016).

By contrast, in non-tonal languages (such as Turkish, Greek, or Spanish), whistles transpose primarily the spoken amplitude and frequency dynamics produced in the acoustic resonances of the front oral part of the mouth – where whistling is also produced – and thus corresponding to the upper part of the timbre of spoken speech. During the whistled articulation, the whistle is captured by the resonance frequency of the cavity situated between the smallest hole in front of the mouth and the smallest hole between the tongue and the palate (i.e., approximately corresponding to the middle of the tongue). When referring to spoken modal speech this would generally include what is called in linguistics the second formant cavity and eventually another neighboring cavity, but the correspondence is not direct because in whistling this cavity is smaller and more constrained than in modal speech. Note that a formant is a concentration of energy produced by a resonance cavity and made of several harmonics of spoken speech. For front vowels, such as /i/, the whistle jumps to the cavity corresponding to the third formant, particularly in vowels which already have close spoken formants 2 and 3 in this area (Shadle, 1983; Meyer, 2015). Mid vowels, such as /e/, are mostly transposing the second formant of modal speech. For several back vowels, such as /a/ and /o/, spoken formants 1 and 2 are close and thus corresponding whistles may sometimes transpose the dynamics of formant 1 due to the reduced articulatory space remaining to produce a whistle (Meyer, 2008; Meyer et al., 2017).

**Figure 9:**
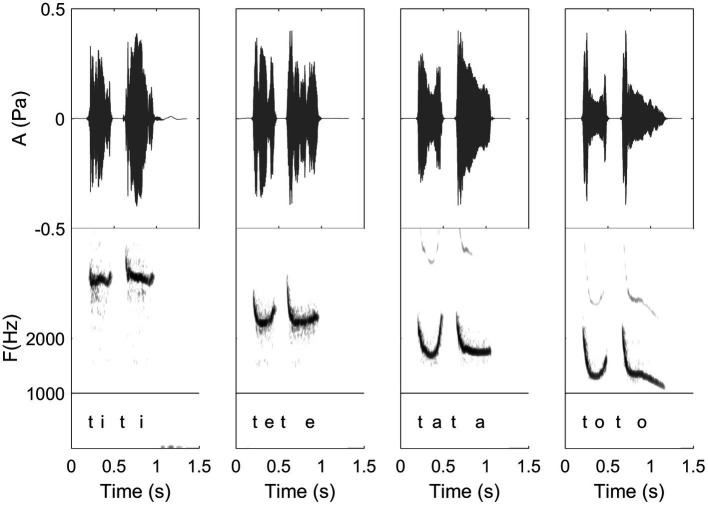
Waveform and spectrogram of /titi/, /tete/, /tata/, and /toto/ as articulated by a Spanish whistler of the Canary Islands. These illustrate first the four different levels of vowels of a same whistler, but also the coarticulation with /t/ of these different frequency levels (see detailed explanation in “General description of dolphin and human whistled communication systems;**”** Recordings and edition by Julien Meyer, listen to sound extracts in Meyer and Diaz, 2021).

**Figure 10:**
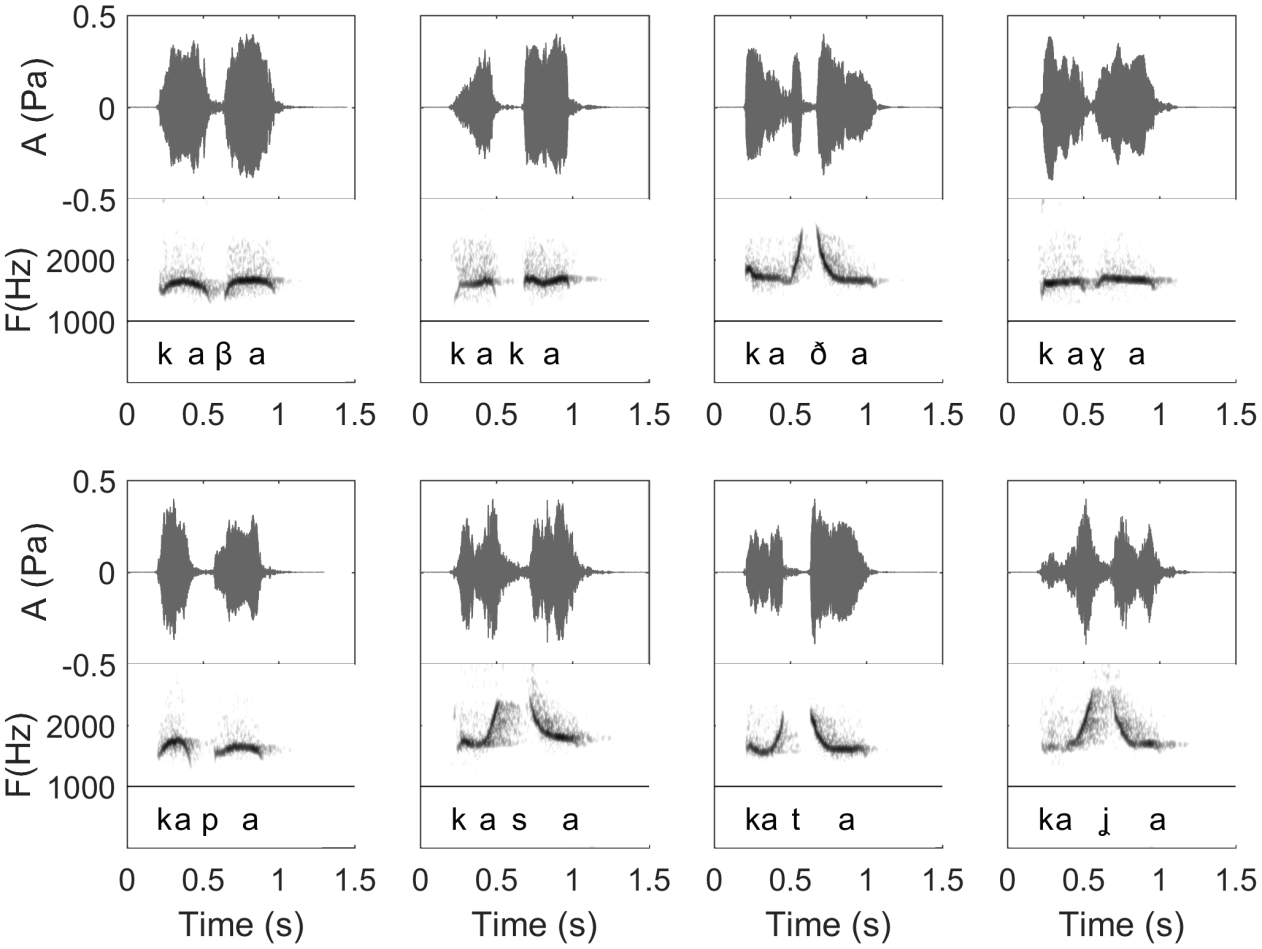
Waveforms and spectrograms of 8 different Spanish words in their whistled form, with the phonetic transcription corresponding to how words were pronounced in spoken speech (International Phonetic Alphabet). From left to right, beginning in the upper line, words are as follows in Spanish orthography: “caba,” “caca,” “cada,” “caga,” “capa,” “casa,” “cata,” and “calla.” This figure shows how the 8 different consonants are encoded differently in whistled speech, resulting in a fine combination of amplitude and frequency modulations defining different frequency profiles: continuity profiles between the two vowels, steepness of the attack of the consonants, and consonantal gap duration in the amplitude envelope. (Recordings and edition by Julien Meyer, listen to sound extracts in Meyer and Diaz, 2021).

In both types of transposition strategies (pitch and formants), the different **vowel qualities** (e.g., /i/, /e/, /a/, /o/, and /u/) or **phonemic tones** (e.g., high, low, and rising) of the spoken languages are whistled at different relative frequencies [see for example Figures 2, 9 where the Spanish /i/ is acute, /o/ is low, and /e/ and /a/ are in between with /e/ higher in frequency than /a/; see Meyer (2021) for details on different languages]. In parallel, in all languages, whistles conform to the way these tones (in tonal languages) and vowel qualities (in non-tonal languages) are influenced by the articulation of surrounding consonants, termed coarticulation in human speech production (Conway et al., 2010) and eventually by other prosodic aspects, such as stress. The **consonants** of spoken speech are created by air blockages and noise sounds formed by the passage of air through the throat and mouth, particularly the tongue and lips. As whistled speech articulation tends to approach the same process, except that it is done in a more closed mouth and without the vibration of the vocal folds, it also results in changes of the airflow which imitates the continuous/interrupted character and/or the celerity of an acoustic attack/release when whistlers try to render the different articulation manners of spoken consonants. Whistled consonants are always produced by performing fine combinations of frequency and amplitude modulations of the whistled frequencies that surround them (which correspond to the tones and vowels of the nucleus of the syllable), reproducing some key phonetic aspects of their spoken equivalent. Thus, between two syllables, whistled consonants are represented by either continuous, near-continuous, or interrupted modulations of the whistles. For example, when the amplitude modulation shuts off the whistle to emulate rapid amplitude modulations of modal speech, whistled consonants are characterized by silent gaps (such as /t/ or /p/ in Figures 1, 2, 9, 10). Signal **segmentation** inside words is thus made by consonants. In whistled tonal languages, it is more the rear part (larynx and laryngopharynx) of the spoken consonant articulation that is imitated by whistling, whereas in non-tonal whistled languages it is more the front part of the spoken articulation that is whistled (front oral cavity).

Finally, human whistlers commonly use a form of **phatic communication** (Jakobson, 1960) to tune to a channel in frequency and to open the conversation by beginning a sentence with a call, such as “aaaa” or “oye” (both are common in Canary Islands, see Classe, 1956; Busnel and Classe, 1976; Rialland, 2005; Diaz, 2008) or “oooo” [common in Atlas, the village where whistled Bearnese of the Pyrenees was documented by Busnel et al. (1962)] or simply a rising tone (observed in some Tamazight villages of the Moroccan Atlas). These initial calls are often followed by the name of the person to which the sentence is addressed. Sentences also often begin in the Canary Islands with common words, such as “mira” (meaning “look” or “pay attention”). The common use of the words *hello* or *hi* in spoken English is another example of such phatic communication in that it serves to get the attention of the recipient and identify the sender. In whistled speech, the exact words or simple interjections that are used vary depending of the language but also vary with the place as each village has its own preferences of uses.

## Dolphin Whistle Communication Acoustic Structure

Studies of bottlenose dolphins and other dolphin species in the wild and in aquaria indicate they have large and complex whistle repertoires (Dreher, 1961; Dreher and Evans, 1964; Kaznadzei et al., 1976; McCowan and Reiss, 1995b) and some results suggest whistle repertoires contain some higher-order internal structure (McCowan et al., 1999). For example, dolphin species, such as the bottlenose dolphin, Atlantic spotted dolphin (*Stellena frontalis*), and saddle-back dolphin (*Delphinus delphis*), to cite some of the best studied species, emit whistled signals that are often produced in whistled sequences that acoustically resemble successions of human whistled short words transposed to higher frequencies (see Figures 2–4, 7). There is evidence that the sequential order of whistle production may be an important feature of these cetacean communication systems (McCowan et al., 1999, 2002). Biphonation, the production of two simultaneous sounds by an individual, has been reported in cetaceans (Tyack and Miller, 2002; Kaplan et al., 2018) as well as in a range of other mammals. Kaplan et al. (2018) reported the production of bitonal and burst-pulse contact calls (signature whistles) by Atlantic spotted dolphins and suggested such biphonal components may serve to convey additional information as to identity, age, or other factors to conspecifics. Whistled frequency contours vary according to the species and range greatly in frequency parameters (Kaplan and Reiss, 2017).

In attempts to decipher dolphin whistle communication, early studies as well as contemporary studies have traditionally approached the problem of segmentation and categorization by using a small number of human-defined whistle categories (e.g., whistle contour and intervals of silence between contours; Dreher and Evans, 1964; Caldwell and Caldwell, 1965; McCowan and Reiss, 1995b). The repetitive characteristic of signature whistles and the predominance of their use relative to other whistle types as discussed in “Dolphin whistle communication” has suggested that they may differ from other whistle types within dolphin repertoires. This informed the development of a method for the identification of signature whistles in free-ranging dolphins termed (SIGID) that is based on the temporal production of these repetitive calls, the inter-whistle or inter-loop intervals of the calls, and the intervals of silence within these repetitive call sequences (Janik et al., 2013). It has been further suggested that such variations may be used by dolphins in differentiating between individuals and populations (Esch et al., 2009; Janik et al., 2013). Variation and categorization of signature whistles have been generally determined by the visual inspection of spectrograms or automated categorization (Buck and Tyack, 1993; McCowan, 1995; Esfahanian et al., 2014) of whistle contours, which vary to different degrees, between individuals and populations. Traditionally, the measurement of acoustic parameters of whistles includes start, end, minimum, or maximum frequency measurements.

## Experimental Approaches to Investigating Dolphin Whistle Functional use and Perception

Past experimental studies have been conducted to investigate and gain insights into the communicative and vocal learning capabilities of dolphin and their perception of whistles. In this section, we discuss two specific studies and the results that are relevant to our understanding of the structure of dolphin whistles.

### Using an Artificial Whistled Derivative of Human Speech to Teach Dolphins an Acoustic Code and Test Their Perceptual Capabilities

One experiment of particular interest for our paper was inspired in part by Busnel’s proposal suggesting the potential relevance of human whistle languages to the study of dolphin communication and his idea that a derivative of a human whistled language could be used to teach dolphins an acoustic code. It was conducted by Batteau and Markley (1967) from 1964– to 1967 to investigate whether they could develop a whistle-like artificial language that could facilitate communication between humans and bottlenose dolphins. This original electronic whistled system consisted in the automatic transformation of spoken English words and nonsense words into whistles. It was based on two artificial transformations impossible in human whistled languages. Firstly, they removed frequencies above 1kHz from the spoken signal. By doing so, they artificially excluded speech information important for phoneme identification in frequency (see research on low pass filtering at 1kHz in Scott, 2001; Baer et al., 2002). Secondly, they used this filtered signal to generate a frequency modulated signal with constant amplitude [see appendix A of Batteau and Markley (Moshier, 1967)], whereas human whistled speech tends to imitate the spoken speech amplitude envelope dynamics even if it simplifies it (see “Human whistled speech acoustic structure: encoding and perception” and Figures 1, 2). Although this methodology of spoken-to-whistle transformation departed from the suggestion of Busnel (1966) to follow the dynamics of a natural whistled language signal, it had the advantage to filter and transform English language phonemes into whistle contours similar in structure to the dolphin’s natural whistled sounds.

This pioneering experiment investigated 1) if dolphins would respond to human speech sounds electronically transformed into whistles, 2) if the dolphins would distinguish between frequency modulated whistles that were derived from the articulated voiced sounds primarily represented by vowels (e.g., several of the artificial nonsense words pronounced by the experimenters in the study, composed of English language vowel and consonant sounds, differed only in vowels – such as /bip/, /baiep/, /baep/, to cite only a few – to determine whether dolphins could differentiate their whistled electronically transposed differences when only the vowels varied), and 3) if dolphins could imitate the modulated whistles.

Note that the whistles presented to the dolphins were in the 5–19.5kHz range to better match the frequency range of bottlenose dolphin whistles. Note also that this voice-to-whistle translator was a one-way system but the ultimate goal was to develop a two-way vocal system to mediate communication between humans and dolphins by inversely transforming dolphin whistle imitations of these sounds back into human words. But this was not pursued as Batteau died before this could be realized (Busnel and Classe, 1976).

Batteau and Markley (1967) reported that through the use of standard operant techniques, two bottlenose dolphins, a male named Maui and a female named Puka, were trained to respond to different whistled sequences that represented mostly 4-word object-action commands structured as (dolphin name – do – object action – go) in which the words for “object action” were represented by one or two whistle(s) and the training set included commands, such as “hit ball with pectoral” (represented by the artificial word /bip/) or “jump out of water” (/jump/), “roll over” (/uweiap/), “swim through hoop” (/baiep/), “make sonar sound” (/baep/), and “raise the fluke” (/beiap/). In the anticipation of creating a bilateral communication system in which the dolphins could productively use the same whistles, one of the dolphins was also trained to imitate the four electronically generated whistles corresponding to the four words /bip/, /baep/, /baiep/, and /uweiap/ when listening to sentences with the following structure: (dolphin name – repeat – object action – go). All in all, it was reported that *“a channel of communication was established which provided necessary transformations of acoustically carried information”* (Batteau and Markley, 1967, p. 78). The authors concluded that *“a basis for the development of a language between man and dolphin has been established”* (*ibid.*, p. 80) basing such an optimistic conclusion on the fact that animals learned to respond behaviorally to these complex commands expressed as sequences of acoustic signals that were direct whistled transformations of simple human spoken phrases.

Two other findings reported in the Batteau and Markley study of particular relevance to this paper and to the deciphering of dolphin whistled communication are as: 1) dolphins could comprehend a command even if part of the sound of the object-action word was missing (/bei/ instead of /beiap/, for example, given that no other invented word in the experiment began by /bei/). This is termed phonemic restoration in human language (Conway et al., 2010). And 2) both dolphins responded correctly when two trainers gave spoken commands transformed into whistles through two different electronic transformation systems (based on the same principle) and despite individual differences in the trainers’ whistled production. This means that the information carried by the invented words and sentences persisted and was transmitted independently of the variation between individual characteristics in the speakers’ voices (or more precisely what remained of this in their electronic transformation). These two points highlight essential features of acoustic languages.

### Affording the Dolphin Degrees of Choice and Control Over Whistle Exposure as a Means to Investigate Vocal Imitation, Learning, and Whistle Perception

In spite of the several advanced technical tools recently developed to analyze bioacoustic signals, how dolphins perceive, segment, and organize their whistles within their interactions remain unclear. We are still far from understanding much about the detailed structure of individual whistles and the temporal organization of dolphin whistle sequences (e.g., are there predictable rules of combination) and the eventual semantic or pragmatic function of whistle sequences during social interactions. What do we really know about how information may be encoded and represented in dolphin whistles based on what we perceive psychoacoustically and visually from spectrographic and other visual representations of dolphin whistles? For example, what are the smallest meaningful units of sound? Are dolphin whistles composed of smaller units of sound analogous to phonemes in human spoken or whistled languages? Are dolphin whistles combinatorial?

On occasion, we get glimpses of possible encoding schemas. In another study, two young captive born male bottlenose dolphins spontaneously imitated novel computer-generated whistles and produced them in behaviorally appropriate contexts [e.g., during interactions with corresponding toys (e.g., balls, rings, or activities; Reiss and McCowan, 1993). Notably, the dolphins began producing novel *combinations* (e.g., ring-ball and ball-ring), in the form of one continuous whistle composed of the two discrete computer whistle, *ball* and *ring* (see Figure 7) and emitted them when engaging in a novel game they created, *double toy play* in which they held a ball and ring in their mouths simultaneously, tossing them into the air and re-catching them in their mouths. The use of the novel combination whistles increased in frequency during the second year of the two-year study. The dolphins were never exposed to two whistles combined by the computer. In this case, it was possible to discern the composite or combinatorial structure of these novel whistles thus providing a clue to the possible structure of dolphin whistles.

The combining or merging and splitting of calls are not at all well understood in dolphins or in other animals. However, evidence for combination calls has been reported in some other species. This has led to much interest in possible parallels between features of human language and patterns in the ways other species, such as elephants, primates, and birds, may combine or merge their calls (Hailman and Ficken, 1986; Arnold and Zuberbühler, 2006; Suzuki, 2014; Engesser et al., 2015, 2016, 2019; Hedwig et al., 2015; Pardo et al., 2019; Tchernichovski et al., 2021). In addition, because the dolphins were not trained to imitate the computer-generated whistles, it was possible to document which acoustic features were salient during the vocal learning process. The dolphins’ facsimiles of the model sounds revealed that they preserved the whistle contour of the model sounds and produced both absolute matches of the time and frequency parameters and signals expanded or compressed within in the frequency-time domain. Such observations give ground to the possibility that the information dolphins encode in whistles are different and more complex than we previously thought. Although we are gaining insights into the possible acoustic features that may be important to dolphins, we are in the infancy of the decoding process. As we will discuss in the next section, these are important features in human speech and human whistled languages. Words vary in acoustic parameters but information is still conveyed. Variations in time-frequency domains in the acoustic communication signals by humans and other species can also convey additional important information associated to – for example – individual identity, group membership, sex, age, emotional state, and physiological state (e.g., Marler and Slabbekoorn, 2004; Dellwo et al., 2007; Pell et al., 2009; Khanna and Sasikumar, 2011; Briefer, 2012). In light of not possessing a Rosetta Stone or a cryptographer’s crib to aid us in deciphering how information is encoded in dolphin whistled communication, we propose that a deeper exploration of how information can be encoded in whistle form as evidenced in human whistle languages may provide important insights to inform the analysis of dolphin whistles.

## Technical Approaches for a Comparative Analysis of Biological Whistles

The application of similar bioacoustics measures and algorithms to dolphin whistles and human whistled speech is possible due to several common aspects between these two whistle systems. As shortly noted above, in the majority of studies of dolphin whistled communication, multiple variables and acoustic parameters are measured – either visually, semi automatically, or automatically – including whistle duration, the number of whistles in a bout of repetitive or differing whistle contours, the number of inflections, start and end frequencies, minimum and maximum frequencies, and ratio of start to end frequency. Whistle boundaries are determined based on the continuity of energy and how it is bounded by intervals of silence. The same acoustic parameters are relevant to measure on human whistles, but as we have previously discussed, other linguistically germane points are considered, which not necessarily correspond to parameters commonly taken into consideration for the dolphins, such as point of median duration of a whistled vowel, for example. Other purely acoustic specificities characterize human whistles when compared to dolphin whistles: They have stronger harmonics and a larger bandwidth relative to their fundamental frequency. When whistling, some flow noise is also generated in humans. This requires an adaptation of the tuning of the detection and frequency estimation parameters (Figure 4) but does not require drastic algorithmic change.

A common problem in the study of dolphin whistle communication and other animal whistled vocalizations is determining the similarity of acoustic signals produced by the same individual, by members within the same social group, and between social groups. For example, as previously mentioned, it was shown that dolphins spontaneously imitated novel computer whistles and their productions of whistle facsimiles of the model sounds revealed that they matched the absolute acoustic parameters of the models but also expanded and compressed the temporal and frequency domains while maintaining the whistle contour. This finding suggests the saliency and potential importance of contour and the plasticity of the temporal and frequency domains (Reiss and McCowan, 1993) and has had significant implications for the analysis of dolphin vocal repertoires. Studies have used time warping also called normalization techniques for the analysis of whistle similarity within dolphin repertoires (Buck and Tyack, 1993; McCowan, 1995). Time-warping techniques have the advantage to allow for the comparison of signals that may differ in absolute acoustic parameters by comparison with relative changes in frequency over time.

Machine learning including unsupervised machine learning offers new perspectives of parallel applications to dolphin and human whistled signals in which they have not been extensively applied yet. Such tools have already had a profound and lasting impact in all of biological data processing all the more as the early and sustained interest of telecom and internet companies to develop speech recognition made machine learning particularly impactful in bioacoustics (Bianco et al., 2019). Because whistled signals have the advantage to be composed of distinct curves in the time-frequency plot of a spectrogram, machine learning can be used to recognize details of such curves or to match them to templates, which is a well-developed area (Tchernichovski et al., 2001). For example, in Gardner et al. (2005), the whistles of canaries were analyzed through an algorithm that first traced the spectrogram contours as individual curves and then applied time-warping techniques to analyze how distinct a given recorded song was from a collection of templates. Because of the similarity between canary, dolphin, and human whistles, this general program is of evident applicability for our case.

Other approaches involve more general machine learning frameworks in which the explicit separation of features is learned. While less sensitive to details, they may be more resilient to noise in recordings in the wild (Kohlsdorf et al., 2016; Jansen et al., 2020; Allen et al., 2021). Similar machine learning approaches have also recently been applied to the bioacoustically relevant case of localizing where a sound came from in a complex acoustical environment (Woodward et al., 2020). Such advanced methods are relevant for the environments that foster dolphin whistled communications and human whistled languages because they are necessary in environments where sound is obstructed by obstacles or where there are massive reflections (reverberation).

Many of these approaches commence by using a spectrogram and treating it as an image, applying image recognition techniques for learning. However, it is well established that the spectrogram *per se* has intrinsic resolution limitations that can be overcome for specific signal classes; two methods that have been used are spectral derivatives (Tchernichovski et al., 2001) and reassigned spectrograms (Gardner and Magnasco, 2006). For example, the reassigned method uses phase information present in the signal to track fast temporal modulations of frequency to high accuracy and thus reveals subtle details of modulation in real recordings that are otherwise difficult or impossible to highlight with classical spectrograms. Studies on automatic and behavioral human speech recognition have shown that details associated with phase information are important in certain circumstances. Although they may not be needed in simple listening tasks and/or at short time scales (e.g., Liu et al., 1997), they are critically needed in challenging tasks (i.e., in noise or with competing speakers, Zeng et al., 2005) and their importance increase as time scales of listening or of analysis increase (Liu et al., 1997; Alsteris and Paliwal, 2005). As suggested by Oppenheim et al. (1979), this happens because phase information associated to frequency modulation highlights the relative location of salient events characterized by rapid frequency variations in syllable edges (such as consonants associated to quick openings, closings, constrictions, and coupling/decoupling of oral cavities).

For dolphin communication, methods of reassigned spectrograms begin to be applied to whistles because their production mechanism has been shown to be able of fine control (see “Dolphin whistle communication”; Reiss and McCowan, 1993). An example of application from Gardner and Magnasco (2006) is available in Figure 6 where rapid jumps in the frequency modulated line of the signal are visible. Such methods could be also applied to human whistles for comparison and for tracking rapid whistled changes that may be perceptually relevant by the human ear but not sufficiently well represented in classical spectrograms. Whistled speech which supposes a challenging perceptual effort, and which is efficient for intelligibility principally in large scale sentence contexts preserves frequency and amplitude modulations from the spoken signal. All these aspects justify why such type of new approach could help to analyze the acoustics/phonetics resulting from the perceptual insight that whistled speech produces into spoken language structures and psycholinguistic processes (Meyer et al., 2017).

Finally, one alternative automatic whistle analysis method completely independent from spectrographic representations of audio files has been developed on dolphin whistles and also already tested on human whistled speech signals (Johansson and White, 2011). It is based on detection and frequency estimation with adaptive notch filters, and it was shown to be applicable to real-world whistle recordings of different characteristics recorded in different settings.

## The Complexity of Human Whistled Speech Illustrated

In this paper, the complexity of the whistled speech signal in relation to its linguistic content is presented more in details for Spanish language because it is the most studied whistled language, but also because the Spanish language pertains to the category of non-tonal whistled languages, the category within which English – the language used as a basis in the Batteau and Markley (1967) report – would fall if it was whistled (note that it is completely possible to whistle English, see examples of audio track of simple sentences provided in Meyer and Diaz, 2021). **Coarticulation** is specifically shown for 8 different consonantal contexts coarticulated with the vowel /a/ on each side (VCV contexts; see Figure 10). More particularly, effect of coarticulation with consonant /t/ appears for the four different vowels /i/, /e/, /a/, and /o/ in Figure 9. An explanation of what happens in terms of articulation is as follows: the “target” toward which the tongue tends to point in order to articulate each consonant corresponds to a specific area of the palate called “locus” of articulation. For /t/, it corresponds to the front part of the palate (just like for spoken speech). When the tongue moves up to this area, the acoustic chamber in the front oral tract is reduced and the whistle thus rises in frequency. The resonance of the front vowel /i/ occurs in the same area, which explains a lesser frequency modulation than with /e/, /a/, and /o/ (as the recordings show in Figure 9). The lower the frequency of the vowel, the more the tongue is retracted in the mouth, which increases the resonance cavity (again as in spoken Spanish or English) and thus lowers the frequency of the whistle. For each of the eight consonants presented in Figure 10, an explanation based on similar considerations can be provided for the associated frequency modulations. **Duration:** some linguistic aspects might be encoded in the relative durations of sounds and silences (the relative aspect is important to let people use different speech rates, for instance). This applies to both vowels and consonants. For vowels, for example, this is the case in Spanish for the “tonic stress” as shown in Figure 8, where the Spanish word “bebé” (meaning “baby”) is encoded differently than the word “bebe” (meaning “he drank”) through the relative duration of the vowels, imitating exactly what is done in spoken Spanish [note that other languages encode three distinct durations of vowels that are mirrored in their whistled form, such as in Siberian Yupik (Meyer, 2015, Chap. 7)]. Note that whistlers tend to articulate slower when communicating at very long distances and prolongation is mostly made on vowels, and especially stressed vowels. For consonants, duration differences were previously measured in the difference of silent duration between voiced and unvoiced consonants positioned between two vowels (Rialland, 2005; Meyer, 2015) see, for example, here an illustration of such differences in the pairs of Spanish words “caba/capa,” or “cata/cada” in Figure 10. There are even other languages in which more consonantal duration categories exist, such as for geminate consonants in Berber Tashelhiyt language of Moroccan Atlas encoded with an even longer silence (Ridouane et al., 2018). **A certain tolerance to variability** not due originally to linguistic reasons applies in all parameters of speech across different whistlers (Figure 2), repetitions of the same word (Figure 8), or different techniques (bilabial whistle, finger whistle…). Moreover, at word boundaries, there is also a tolerance to variability linked to whether the speaker connects the words or not, just like what happens in spoken speech. A very striking example of such a case is presented in Figures 2, 4 where the first syllables /bena/ are effectively pronounced /bena/ by the first speaker (where /n/ is continuous between /e/ and /a/), whereas the second speaker says /ben.a/ with a clear pause between /en/ and /a/.

## Discussion and Conclusion

In this paper, we extensively revisited the current knowledge on human whistled speech, a natural modality of human communication presents worldwide across a great diversity of languages and its potential relevance to the study of dolphin whistle communication. For human listeners, language sciences in general, and scientists studying non-human animal communication, whistled forms of human languages provide a provocative and alternative point of view on what can be encoded in a simple frequency and amplitude modulated whistled message. Strikingly, based on what is now known about the production, perception, and the nature of the human whistled speech communication, complex information can be encoded in what may appear to be simple calls.

We also revisited literature on dolphin whistle communication from field studies and observational and experimental studies conducted in aquaria. The cognitively complex and highly social dolphins have demonstrated a high level of complexity of their productive, computational, and perceptual capacities including their proclivity for vocal imitation and vocal learning, evidence for spontaneous combinatorial production of combined elements and behavioral concordance in their use, and comprehension of human generated grammatical “sentences. In this context, we suggest, based on what is also now known about the representation of complex information in human whistled speech, that more complex information may be encoded in dolphin whistled communication than previously thought and shown. We are not proposing, though, that the whistles of dolphins (or other complex whistling species) encode similar types of information as human whistled languages but rather that the way information is structured in whistled speech may inspire research on dolphin whistled communication.

### Exploring Dolphin Whistle Structure and Segmentation

A major challenge to decoding dolphin whistle communication is that we lack an understanding of the structure of dolphin whistles. Are they comprised of smaller subunits? Might they be combinatorial (e.g., in comprised of two discrete units) similar to the combination whistle in Figure 7 reported by Reiss and McCowan (1993)? Also, little is known regarding the temporal organization of dolphin whistles. Thus, advances in deciphering dolphin whistle repertoires will ultimately require elucidation of the structure of the smallest segments of sounds (akin to phonemes in humans) or meaningful sounds (akin to the morpheme) and how they are structurally and temporally organized and how they are used pragmatically (functionally) in social transactions between dolphins. Although most approaches to classifying and categorizing dolphin whistles generally segment the vocal sequences by relying on the intervals of silence between whistle elements (Dreher and Evans, 1964; Caldwell and Caldwell, 1965; McCowan and Reiss, 1995b; Esch et al., 2009; Janik et al., 2013), it remains unclear where dolphins perceive the boundaries between elements. Such an approach, although the most accessible way to presently observe and analyze dolphin whistled signals may not accurately represent whistle boundaries, the finer structure of whistles nor their temporal organization is in sequences.

Of particular relevance and potential importance to the analysis of dolphin whistled communication, is how human whistle sequences are represented spectrographically and how, in parallel, they are segmented by the listeners. Looking at spectrograms of human whistled speech, a naïve observer might also assume that each whistle bounded by intervals of silence is a specific whistle and would rely on this means of categorization. However as is evidenced in human species, this approach does not match how words are segmented psychoacoustically by the human whistler or those listening and decoding. Just as for spoken modal speech, in representations of human whistled phrases and sentences, segmentation between words (word boundaries) is not always clearly demarcated by inter-word intervals of silence (as seen in Figure 2). It is well known that speech *segmentation* during speech perception is largely a psychoacoustic process (Davis and Johnsrude, 2007) and the listener’s knowledge about language facilitates placing word boundaries in appropriate locations and making sense of a stream of sounds. This holds true for the perception of human whistled speech as well. As previously discussed, what may appear as one continuous whistle may be comprised of more than one word or a word and the beginning syllable of the next word. Interestingly, segmentation of acoustic sentences, categorization, and parsing of words are less clear for whistled speech than is the case for spoken speech. This because in whistled speech there are less acoustic cues available to help segment the consonants from their surrounding vowels and there are closer similarities between some types of consonants and vowels in each language.

### The Perspectives Opened by Behavioral Psychoacoustic Tests

As discussed earlier, psychoacoustic behavioral experiments have addressed the issue of intelligibility of words and phrases in human whistled speech showing that even if it eliminates canonical acoustic correlates of phonemes from the spectrum, it keeps essential and sufficient speech signal modulations in amplitude and frequency to enable high intelligibility levels of around 70% for words and 80–90% for sentences. This demonstrates the amazing productive flexibility present in human speech relying on the fact that the referent modal spoken speech form is encoded in a large amount of acoustic cues that contribute to compensating for signal distortions and noisy interferences (Assmann and Summerfield, 2004). But, such a simplification of speech encoding is only possible because humans also have a great capacity in terms of perceptual flexibility, i.e., the ability to recognize a stimulus as belonging to a category even if partly deviant from the acoustic form to which they are used (i.e., speech in noise and foreign accents). Thus, strikingly, the whistled natural type of speech exploits the fact that speech recognition by human listeners is very resilient: It remains possible even after large amounts of the signal have been removed because our human perceptual and cognitive systems are adapted to overcome speech degradations (Miller and Licklider, 1950; Remez et al., 1981; Warren et al., 1995; Arai and Greenberg, 1998; Palmer and Shamma, 2004). Several recognition tests on finer degrees of linguistic cues have also been performed on vowels and consonants in Consonant-Vowel or Vowel-Consonant-Vowel contexts and in various different conditions: with trained human whistlers or naïve human listeners of different language background (Meyer, 2005; Rialland, 2005; Meyer et al., 2017, 2019; Ngoc et al., 2020a,b). The protocols that have been designed for such perceptual studies are of the type of the ones that can be run on simple keyboards (4-AFC, AXB, or same/different tasks, for example). The fact that multiple choice keyboard experiments were already run with dolphins (Reiss and McCowan, 1993) shows that such approaches could be adapted for dolphins. Presenting whistles encoding the complexity of human language to dolphins would represent a unique opportunity to profit from a common acoustic mean of communication to test more abstract representations than what has been done so far. It is necessarily an anthropocentric view, but it is a point of departure.

The relevance of previous experimental studies with dolphins in this domain is also supported by the results of the early study by Batteau and Markley (1967) which advanced our understanding to some degree about the ability of dolphins to perceive and comprehend a spoken-to-whistled transformation. Although the methodology of artificial electronic transformation used by Batteau and Markley resulted in whistled stimuli presented to the dolphins that differed significantly from those of natural spoken languages transformed into human whistled languages (artificially excluding speech information important for phoneme identification (e.g., Scott, 2001; Baer et al., 2002) among which frequencies above 1kHz corresponding to the area of whistled articulation in non-tonal languages], the experiment showed important results. It found that dolphins demonstrated the ability for signal restoration in a way that evoked phonemic restoration in language – a phenomenon in which the brain restores the missing information and the individual reconstructs the referential meaning of the corresponding intact stimuli. This perceptual ability enables individuals to process information that is degraded due environmental noise or signal distortion and we now know that dolphins are able to modify their calls in the face of noise (Fouda et al., 2018), which suggests the ability of dolphins to perceive noise pollution and sound degradation of their communicative signals in their environment. The dolphins were also able to differentiate whistled transformations of words like /baiep/ and /baep/ which differed only in one additional frequency level of /i/ in a continuous whistle, or words like /baiep/ and /beiap/ in which the difference was in inverting the relative frequency levels of /a/ and /e/ in a continuous whistle. Finally, the dolphin’s ability to correctly respond to whistled commands spoken by two trainers and transformed into whistles through two different electronic transformation systems also suggests a tolerance for whistle variability.

### Variability and Coarticulation

How dolphins perceive acoustic variability in the vocalizations of conspecifics and the type of information it may or may not convey is hard to determine. Studies of human whistled languages provide interesting tracks to follow as they have reported that the duration of whistles and relative durations of sounds and silences can convey information (see for example Figure 10). Different speech rates are tolerated and these may even change whistled segmentation between different whistled units (see /bena/ in the beginning of Figure 2 for example). This may be a means of conveying information in dolphin whistled communication as well and needs further investigation. As discussed in the section on dolphin whistle communication, Reiss and McCowan (1993) reported that dolphins transposed, expanded, and compressed their whistles in the time and frequency domain yet the overall whistle contour remained stable. This has important implications for sound analysis programs when tracking and comparing whistle contours of the same and different individuals. Furthermore, in terms of the fine structure of whistles, it is unclear whether information is conveyed in what may appear to us as subtle variations in the whistle contour, such as small steps and small jumps in frequency as seen in Figure 4.

The phenomenon of *coarticulation* is present in human spoken and whistled speech. The documentation of the variability in the acoustic parameters of whistles of individuals could enable an assessment of coarticulation by examining dolphin whistle sequences produced by individuals to determine if there is evidence for variability due to coarticulation (e.g., variability due to the preceding and following whistles). As previously discussed, in a non-tonal language, such as Spanish coarticulation is when vocalic frequencies are influenced by a neighboring sound or syllable.

### Phatic Communication

Finally, *phatic communication* is an important part of human communication whether it is spoken in person, *via* a cell phone or whistled. The phatic function of language serves a socio-pragmatic role to open and maintain communication channels in whistled languages as it does in spoken languages. Words such a “hello” or “bonjour” are such examples. Although aspects of the voice can convey identity and other information about sender, such as sex/gender, physiological state, and nationality/ group identity such phatic expressions are not considered to be communicative in terms of informational content but they function in establishing, maintaining, and managing bonds of sociality between participants (Malinowski, 1923). The use of phatic communication may be more widespread in non-human animals as a similar function may be found in their contact calls. For example, in the case of dolphins, the broadcasting the one’s contact call (e.g., signature whistle in dolphins; Caldwell and Caldwell, 1965; McCowan and Reiss, 1995a,b, 2001; Janik and Sayigh, 2013; Luís et al., 2021) that is thought to convey whistler identity and establish and maintain contact and group cohesion may also serve an additional role as well in opening a channel of communication and focus the attention of conspecifics on information that will follow. This would necessitate the ability to track and attribute vocalizations to specific individuals and examine the types of whistles and the temporal organization of whistles within sequences.

### Conclusion

To conclude, natural human whistled speech can be seen as a kind of intermediate for the analysis and decoding of dolphin whistles. To proceed in decoding at a deeper level than what is already done in dolphin whistle communication requires an understanding of dolphin whistle structure and the temporal organization of whistles within whistle sequences. Thus, we propose that some of the characteristics found in human whistled communication may be worth considering in the analysis of whistled signals of dolphins. Viewing dolphin whistle repertoires through a lens that allow us to focus on the characteristics of the acoustic structure and organization found across human whistled languages coupled with what is known about the dolphins’ own capacities and proclivities may provide new insights and help to develop new analytic tools and approaches that will advance deciphering whistle communication in dolphins. Further applications of information theory to the analysis of dolphin whistle vocalizations as previously conducted by McCowan et al. (1999) offer a promising approach for future comparisons of the informational structure of human and dolphin whistle communication and whistle repertoires. For this, an extensive curated database of whistle sequences produced by single individuals as well as whistle sequences produced during vocal exchanges between two or a few individuals would be optimal. This type of database would also be optimal for future studies to take a deeper dive into the structure and perhaps finer details of individual whistles and their temporal organization in whistle sequences and exchanges. Analytic approaches and tools used to decipher dolphin whistled communication may benefit and advance from the consideration of what is known about how information is encoded in the case of human whistles – for which we have a translation.

## Data Availability Statement

The datasets presented in this study can be found in online repositories. The names of the repository/repositories and accession number(s) can be found at: https://soundcloud.com/user-28976943/sets/meyer-and-diaz-2021-sounds-of-whistled-speech, https://soundcloud.com/user-28976943/sets/reiss-and-magnasco-sounds-of-dolphins.

## Ethics Statement

The studies involving human participants were reviewed and approved by the Comité d’évaluation éthique of SPIN-CNRS and the Council of the Government of El Hierro (Expte 1997/2021, Canary Islands, Spain). The participants provided their written informed consent to participate in this study. The animal study was reviewed and approved by the IACUC committees at Hunter College and The Rockefeller University, the Belize Fisheries and Forestry Departments (Permit Ref. No. WL/1/1/16(28), and the Bahamas Environment, Science and Technology Commission and the Department of Marine Resources. Written informed consent was obtained from the individual(s) for the publication of any potentially identifiable images or data included in this article.

## Author Contributions

JM, DR, and MM conceived and wrote the paper. All authors contributed to the article and approved the submitted version.

## Funding

The collaborative research on dolphin communication by DR and MM was supported in part by grants from the National Science Foundation (grant number PHY-1530544 and PHY-1606535) and the Eric and Wendy Schmidt Fund for Strategic Innovation. The research on whistled Spanish and the fieldwork recordings presented on whistled speech by JM was financially supported by the seventh research program of the European Community FP7 2007–2013 (Marie Curie IIF Grant 630076 developed between 2013 and 2015). Additional whistled speech research was developed and financed in 2020-2021 in the framework of the project GeoTeleLing with the support of the CNRS 80Prime interdisciplinary programme, including for the publication in this open access journal.

## Conflict of Interest

The authors declare that the research was conducted in the absence of any commercial or financial relationships that could be construed as a potential conflict of interest.

## Publisher’s Note

All claims expressed in this article are solely those of the authors and do not necessarily represent those of their affiliated organizations, or those of the publisher, the editors and the reviewers. Any product that may be evaluated in this article, or claim that may be made by its manufacturer, is not guaranteed or endorsed by the publisher.
